# Muddy Skirts

**Published:** 1899-01

**Authors:** 


### Muddy Skirts.

Lady Haberton sees, in the attention at present being drawn to the spread of tubercular disease, an opportunity for again warning her sisters of the danger they incur in wearing long skirts. She is quoted in the *Times,* and as asserting that "she considers tuberculosis is greatly assisted by the ladies who drag their skirts through the filth of the streets." Her charge has stirred the soul of a

*Westminster Gazette* poet, "R. C. R.," who on Monday last produced the following lines:

Life is not a bed of roses,
When the vile tuberculosis
Has the upper hand.
But the *rational*isation
Of the ladies of our nation,
So I'm given to understand,
Will remove its awful terrors,
Due to skirts and *sweeping* errors
In the costume a la mode.
Yet I never had a notion
That the poetry of motion
Gathered microbes from the road.
And the cause of all distress is
Mud that dries upon the dresses,
Harboring a wealth of dirt—
At least Lady H. supposes
This to be the diagnosis
Of the fell tuberculosis.
Why not try the "Bloomer" process
And eschew the poisoned skirt?

—*The Sanitary Record, London.*

